# Identification of piRNA Targets in Urinary Extracellular Vesicles for the Diagnosis of Prostate Cancer

**DOI:** 10.3390/diagnostics11101828

**Published:** 2021-10-03

**Authors:** Qiang Peng, Peter Ka-Fung Chiu, Christine Yim-Ping Wong, Carol Ka-Lo Cheng, Jeremy Yuen-Chun Teoh, Chi-Fai Ng

**Affiliations:** SH Ho Urology Centre, Department of Surgery, The Chinese University of Hong Kong, Hong Kong, China; pengqiang@surgery.cuhk.edu.hk (Q.P.); peterchiu@surgery.cuhk.edu.hk (P.K.-F.C.); christinewong@surgery.cuhk.edu.hk (C.Y.-P.W.); carolcheng@surgery.cuhk.edu.hk (C.K.-L.C.); jeremyteoh@surgery.cuhk.edu.hk (J.Y.-C.T.)

**Keywords:** prostate cancer, PIWI-interacting RNAs, biomarkers, urinary EVs, non-coding RNA

## Abstract

Emerging studies demonstrate that PIWI-interacting RNAs (piRNAs) are associated with various human cancers. This study aimed to evaluate the urinary extracellular vesicles (EVs) piRNAs as non-invasive biomarkers for prostate cancer (PCa) diagnosis. RNA was extracted from urinary EVs from five PCa patients and five healthy controls (HC), and the piRNAs were analyzed by small RNA sequencing. Dysregulated piRNAs were identified and then validated in another 30 PCa patients and 10 HC by reverse-transcription polymerase chain reaction (RT-qPCR). The expressions of novel_pir349843, novel_pir382289, novel_pir158533, and hsa_piR_002468 in urinary EVs were significantly increased in the PCa group compared with the HC group. The area under the curve (AUC) of novel_pir158533, novel_pir349843, novel_pir382289, hsa_piR_002468, and the combination of the four piRNA in PCa diagnosis was 0.723, 0.757, 0.777, 0.783, and 0.853, respectively. After the RNAhybrid program analysis, all four piRNAs had multiple potential binding sites with key mRNAs in PTEN/PI3K/Akt, Wnt/beta-catenin, or androgen receptor pathway, which are critical in PCa development and progression. In conclusion, our findings indicate that specific piRNAs in urinary EVs may serve as non-invasive diagnostic biomarkers for PCa.

## 1. Introduction

PCa is one of the most common male urinary malignancies, with the highest morbidity and mortality among male malignancies in Europe and America [1]. While the use of serum PSA has greatly improved the proportion of patients diagnosed at localized /early stage, there are also problems of overdiagnosis and overtreatment [2]. Therefore, it is clear that there is a clinical need for improved PCa diagnostic biomarkers. Urine is a convenient way to detect biomarkers released from the prostate gland for the identification of PCa. These biomarkers, often referred as liquid biopsies [3], may better reflect the heterogeneity of the tumor than single biopsies. When compared to blood or tissue-based markers, urine-based makers have potential advantages in the assessment of the prostate. Besides being non-invasive and thus large quantities can be collected, exfoliated cancer cells and prostatic secretions can be analyzed.

EVs are heterogeneous membrane-enclosed phospholipid vesicles, ranging from approximately 30 to 1000 nm in diameter [4]. By transferring its content (protein, nucleic acid, metabolites, and lipids) to other cells, EVs are important for intercellular communication and have been shown to be important in PCa development [5]. EVs can be detected in various biological fluids, including urine. Due to the protective effect of the lipid bilayer from enzymatic degradation, biological content within EVs is relatively stable and is a good source for studying potential markers. Among these, urinary EVs non-coding RNAs (ncRNAs) play important roles in PCa carcinogenesis [6]. ncRNAs are generated from non-coding regions that do not encode protein sequences, which account for approximately 98% of all transcriptional output in humans [7]. Recently, PIWI-interacting RNAs (piRNAs) have been identified as a novel class of ncRNAs. They have a length of 26–32 nt and are characterized by 3’terminal 2’-O-methylation [8,9]. Except for transposon repression, piRNAs could post-transcriptionally regulate gene expression through piRNA-RNA interactions in cytoplasm [10], similar to miRNA mechanisms, or epigenetically silence genes. Among all non-coding RNAs, piRNAs are suggested to be the most abundant and diverse small non-coding RNA, being derived from all types of genomic sequences [11]. More than 30,000 piRNA species were described in the human genome, which is more than that of miRNAs (~2000) [12]. piRNAs are also more resistant to oxidation and degradation than miRNAs, due to their 2′-O-methyl (2′-O-Me) modification on 3′ terminal base [13]. piRNAs are named due to their characteristics of exclusive association with the PIWI protein family in which they play regulatory roles, making them more stable than miRNAs due to the protection function of PIWI protein [14]. These characteristics make piRNAs potential biomarkers for cancer diagnosis. 

Growing evidence shows that various piRNAs differently express between cancer tissues and matched normal tissues, and involve a variety of solid tumors and blood system tumors [10]. Several studies also identified that piRNAs are stably present in human blood, and can serve as biomarkers for cancer diagnosis [14,15,16]. Beyond the role of piRNAs as biomarkers, studies have also demonstrated their potential role as therapeutic tools. It has been reported that artificial piRNAs generated by the expression of sense and antisense transcripts were sufficient to induce epigenetic silencing of the target gene in a mouse model [17]. In addition, piRNAs are also involved in the proliferation, apoptosis, metastasis, and invasion of cancer cells, and may be potential prognostic biomarkers in the development of cancer [18]. Thus, modulating the expression of piRNAs may reverse these phenotypes. As for PCa, several recent studies revealed that piRNAs were involved in the proliferation, migration, and invasion of PCa cells by conducting different signal pathways, which may represent a new marker of PCa diagnosis [19,20].

As the largest class of small ncRNAs that are different from long RNAs, piRNAs are not easily degraded and can easily move through cell membrane [21]. This characteristic implies that piRNAs could be detected easily in various body fluids, such as urine. Accumulating studies have reported that miRNAs are contained in EVs and can be used as promising biomarkers for cancers [22,23]. However, the role of urinary EVs piRNAs in PCa diagnosis has not been investigated before. In this study, urinary EVs were isolated from patients with or without PCa. By using small RNA sequencing, potential piRNA targets were shortlisted and then further validated in a separate cohort. We then further analyzed the values of various urinary EVs piRNAs in PCa diagnosis.

## 2. Materials and Methods

### 2.1. Patients and Samples

The study was approved by the Joint Chinese University of Hong Kong—New Territories East cluster clinical research ethics committee (CREC-2018.063). After obtaining written consent, 100 mL urine without prostatic massage was collected from 5 PCa patients as the screening cohort. Another 5 patients with no evidence of PCa, serum PSA < 4 ng/mL, and normal digital rectal examination (DRE), were recruited from our urology clinic as control. Collected Urine was centrifuged at 3000 rpm for 20 min at 4 °C, and the cell free portion was stored at −80 °C for further usage. Another 30 PCa patients and 10 controls (PSA < 4 ng/mL and normal DRE) were also recruited and consented for the validation part of the study. The urine handling process was identical to the screening cohort.

### 2.2. Isolation and Confirmation of Urinary EVs

100 mL cell free urines were thawed on ice and urinary EVs were concentrated with Amicon^®^ Ultra-15 Centrifugal Filter Unit with an Ultracel-3 membrane of 3 kDa (Merck-Millipore, Burlington, MA, USA) at 4000 *g* for 20 min with a swinging bucket rotor. Concentrated EVs were purified with theUrine Exosome Purification Kit (Norgen Bioteck, Thorold, ON, Canada) and resuspended EVs were characterized by TEM and Western blot with EVs marker TSG101 (Abcam, Cambridge, UK) and LAMP2b (Abcam).

### 2.3. EVs RNA Extraction

Total RNA was extracted from the urinary EVs using a Urine Exosome RNA Isolation Kit (Norgen Bioteck) according to the manufacturer’s protocol. The extracted RNA was eluted in 20 µL of RNase-free water. The quantity of RNA was then measured using a Qubit™ RNA HS Assay Kit and Qubit 4 Fluorometer (Termo Fisher Scientifc, Waltham, MA, USA). RNA quality was assessed by an Agilent 2100 bioanalyzer using the RNA 6000 Pico Chip (Agilent Technologies, Middelburg, The Netherlands).

### 2.4. Transmission Electron Microscopy

Freshly isolated EVs suspensions were fixed with 4% paraformaldehyde in 0.1 M phosphate buffer (pH 7.4). A drop of each sample was placed on a carbon-coated copper grid for 20 min and negative staining with 1% uranyl acetate for 1 min. The morphology of EVs was observed under the transmission electron microscope (JEM-1400EX, JEOL Ltd., Tokyo, Japan).

### 2.5. Western Blot Analysis

EVs were lysed with 40 µL of RIPA buffer supplemented with protease inhibitor. Then, 10 µg of total protein were prepared for Western blot analysis. Twenty micrograms of EVs protein were separated on a 10% sodium dodecyl sulfate polyacrylamide gel electrophoresis (SDS-PAGE), and then transferred to a PVDF membrane (Millipore). Membranes were blocked with 5% skim milk for 1 h at room temperature and were immunostained with primary antibody against TSG101 (Abcam) or LAMP2b (Abcam), at a dilution of 1:1000 overnight at 4 °C, and corresponding secondary antibody 1 h at room temperature. The Clarity and Clarity Max ECL Substrates (Bio-Rad) were applied for detecting specific bands on membranes.

### 2.6. Small RNA Sequencing and Sequencing Data Analysis

Small RNA library preparation was carried out by Illumina TruSeq Small RNA Library Preparation Kit, and Amplified libraries were sequenced with BGISEQ-500 technology (BGI-Shenzhen, Shenzhen, China). Small RNAs were ligated to adenylated 3′ adapters annealed to unique molecular identifiers, followed by ligation of 5′ adapters. The raw sequences were filtered to remove tags with low quality, with 5′ contamination, with poly A tails, without 3′ primer, without insertion, or shorter than 18 nucleotides. The obtained clean reads were mapped against piRNABank (http://pirnabank.ibab.ac.in/, accessed on 4 March 2019) to screen and annotate piRNAs using Bowtie. Piano is used to predict piRNAs. This algorithm is based on the support vector machine (SVM) algorithm and transposon interaction information, and the SVM algorithm can also be used in a wide range of species, including human, mouse, rat, fruitfly, and insects. After annotation, the expression level of novel or unknown piRNA was calculated by using transcripts per kilobase million (TPM). Differentially expressed genes (DEGs) were shortlisted when reads number at fold change ≥2 and q-value ≤ 0.001.

### 2.7. Reverse Transcription and RT-qPCR of Urinary EVs piRNA

Urine samples were processed to collect cell free urinary EVs RNA. Extracted RNA was reversed transcribed with TaqMan^®^ microRNA Assay Kit (Thermo Fisher) and TaqMan^®^ MicroRNA Reverse Transcription Kit (Thermo Fisher). A final volume of 12 µL for each reaction underwent RT using a QuanStudio 7Flex system at 16 °C for 30 min, 42 °C for 30 min, followed by a final step of 85 °C for 5 min. To increase sensitivity and specificity, a 12-cycle preamplification step was included. Briefly, an equal mix of all 20× TaqMan miRNA assay probes was prepared for each reaction and diluted to 0.2× with 1× Tris-EDTA buffer (pH 8.0). Each sample contained 12.5 µL 2× TaqMan PreAmp Master Mix, 3.75 µL of diluted TaqMan assay probe mix, and 8.75 µL of multiplexed cDNA product. The preamplification cycling condition was set as follows: 1 cycle of 95 °C for 10 min, 55 °C for 2 min, 72 °C for 2min, 12 cycles of 95 °C for 15 s and 60 °C for 4 min, 1 cycle of 99.9 °C for 10 min were run on a QuanStudio 7Flex system (Thermo Fisher). The resulting reaction products were diluted 1:9 with nuclease-free water to a final volume of 100 µL. At last, 1.5 µL of the diluted preamplification product was added to 10 µL 2× TaqMan Advanced PCR Master Mix (Thermo Fisher), and 1 µL of each individual 20× TaqMan primer/probe assay. The PCR cycling condition was set as follows: 1 cycle at 95 °C for 20 s, 40 cycles at 95 °C for 1 s, and 60 °C for the 20 s. All reactions were performed in duplicate. All specific primers and probes of piRNAs were custom designed from Thermo Fisher Scientific, and housekeeping snU6 was commercially available from Thermo Fisher Scientific. Relative piRNA expression levels were determined by normalizing to snU6 using the 2−ΔCT method. Specific primers used in RT-qPCR are detailed in Table S1.

### 2.8. Identification of Key piRNAs Potential Downstream Mechanisms

Effective mRNA: piRNA interaction requires strict base pairing within 2–11 nt at the 5′-end of piRNA, and less stringent base pairing within 12–21 nt [10,24]. Based upon piRNA:mRNA sequence complementarity, the RNAhybrid algorithm was used to predict the potential target genes of key piRNAs (*p* < 0.05, binding free energy < −20 kcal). The potential binding effect between the dysregulated piRNAs and key molecules involved in PCa pathways were explored to further understand the potential regulatory mechanism of piRNAs in PCa [25].

### 2.9. Statistical Analysis

Statistical analysis was performed by SPSS for Windows software version 23.0. The non-parametric Mann–Whitney test was used to test for differences in piRNA expression between groups, with a *p*-value of < 0.05 being statistically significant. Receiver operating characteristic (ROC) curve analysis was plotted using each piRNA expression value to estimate the diagnostic values of the urinary EVs piRNAs. AUC with 95% confidence interval (CI) was calculated for each ROC curve.

## 3. Results

### 3.1. Patient Clinical Details

The screening cohort included five PCa patients and five healthy controls. The validation cohort included 30 PCa patients and 10 healthy controls. The baseline characteristics of both cohorts were described in Table 1.

### 3.2. Identification of Urinary Cell-Free EVs and Characterization of Urinary EVs RNA

**Figure 1:**
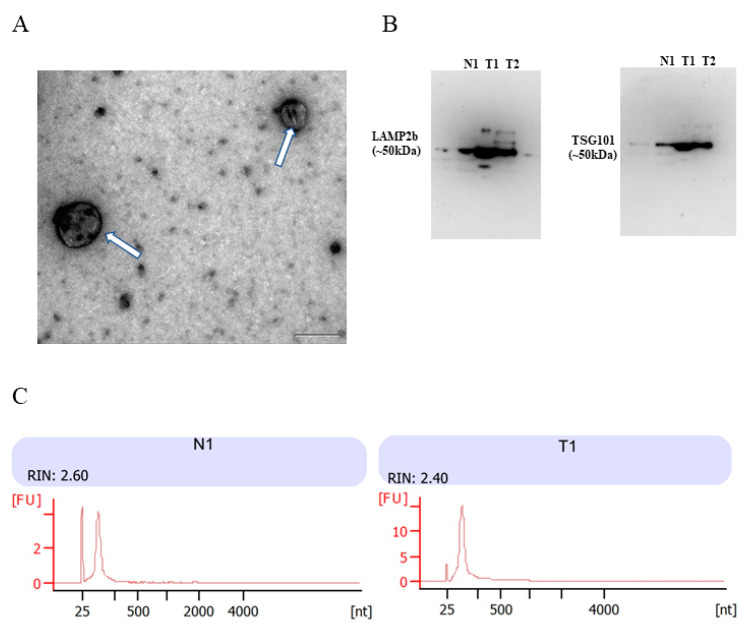
Characterization of EVs from urine samples. (**A**) Isolated particles were analyzed using TEM, which showed the typical size and morphology. Typical EVs were highlighted using white arrows. Solid black lines represent 100 nm scale bars; (**B**) Western blot analysis of urinary EVs with antibodies LAMP2b and TSG101 from one healthy control (N1) and two PCa patients (T1 and T2); (**C**) the RNA size distribution of urinary EVs from one healthy control (N1) and one PCa patient (T1) was analyzed by bioanalyzer analysis.

To confirm the successful isolation of urinary cell-free EVs, transmission electronic microscope (TEM) was carried out to visualize morphology. From TEM images, the isolated particles have a typical size and morphology of EVs (Figure 1A). To further confirm the presence of EVs, two EV markers TSG101 and LAMP2b were evaluated by Western blot with anti-TSG101 and anti-LAMP2b antibody. Results showed that positive bands around 50 kDa were detected in the particles isolated from one healthy control and two PCa urine samples (Figure 1B). The integrity and RNA size distributions of urinary EVs RNAs were analyzed by the Agilent 2100 Bioanalyzer Instrument (Figure 1C). Results showed that the RNA integrity number (RIN) of urinary EVs RNA samples was around 2.5, and contained a high proportion of small RNAs, which was consistent with the characterization of EVs RNA. The above demonstrated successful purification of EVs from patient urine by ultrafiltration, and RNA extracted from EVs were an adequate quantity and quality for small RNA sequencing.

### 3.3. Small RNA Sequencing of Urinary Cell-Free EVs RNA

To compare the piRNA profiles between PCa and non-PCa patients, small RNA sequencing was done with urinary cell-free EVs from five PCa urine and five non-PCa controls. From the sequencing data, there were 20–26 million total clean tags and approximately 74–97% of the reads were mapped to the genome (Table S2). After annotation and prediction, miRNA occupied 0.4–15%, follow by piRNA 0.09–22.6%. The rest of the reads mapped to rRNA, snoRNA, snRNA, tRNA, and rfam gene together made 0.6–8.34% of the reads. piRNAs were first annotated in the piRNABank database, then predicted with Piano algorithm. Results demonstrated that the total piRNAs counts ranged from 8343 to 39,764, with a median count of 18,332 in our non-PCa sample samples. Whereas, PCa had 3098–60,766 piRNA counts with a median count of 44,581 (Table S3). Among the piRNAs with significantly differential expression levels, we selected 10 piRNAs with absolute value of log2 fold change >2 and q-value < 0.01 as candidates, due to their read counts (Table 2).

### 3.4. Validation of Urinary EVs piRNAs in Another Cohort of Patients

**Figure 2:**
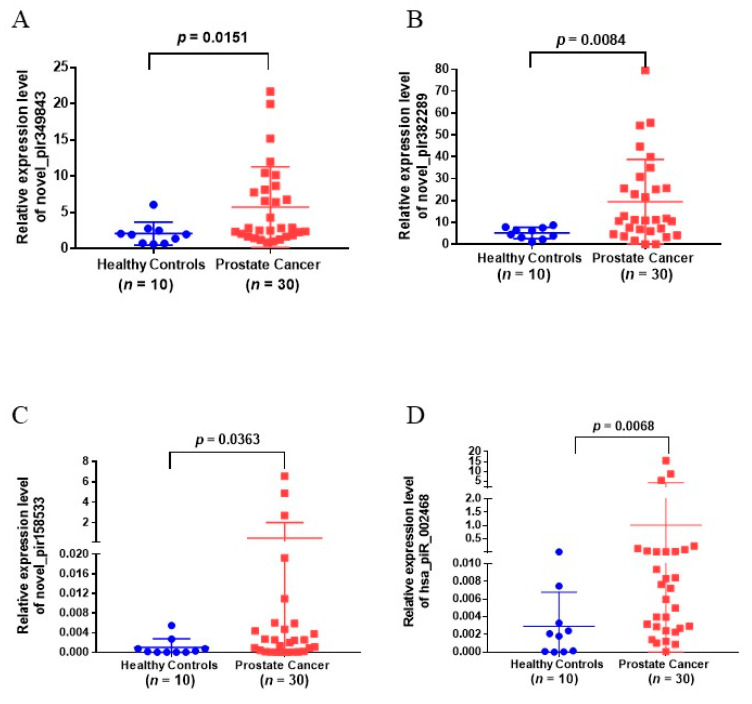
Upregulated piRNAs in validation cohorts. (**A**–**D**) Relative piRNA expression levels of (**A**) novel_pir349843, (**B**) novel_pir382289, (**C**) novel_pir158533, and (**D**) hsa_piR_002468 were examined in urinary EVs of PCa patients (*n* = 30) and healthy controls (*n* = 10) using TaqMan real-time PCR Assays.

To validate the EVs piRNAs identified from small RNA sequencing, 10 candidate piRNAs in urinary EVs were analyzed in a validation cohort of 30 PCa patients and 10 healthy controls by RT-qPCR with TaqMan probe assay. Results showed that novel_pir349843, novel_pir382289, novel_pir158533, and hsa_piR_002468 were significantly upregulated in urinary EVs from PCa patients as compared with non-PCa cases (*p*-value < 0.05, Figure 2, Table 2). However, there was no significant difference in the expression of novel_pir99473, novel_pir99492, novel_pir4470, novel_pir50190, and hsa_piR_001170 in the PCa group as compared to non-PCa cases (Figure S1, Table 2), and novel_pir123744 was not detected in the experimental conditions that were used.

### 3.5. The Diagnostic Value of Putative EVs piRNA Biomarkers

**Figure 3:**
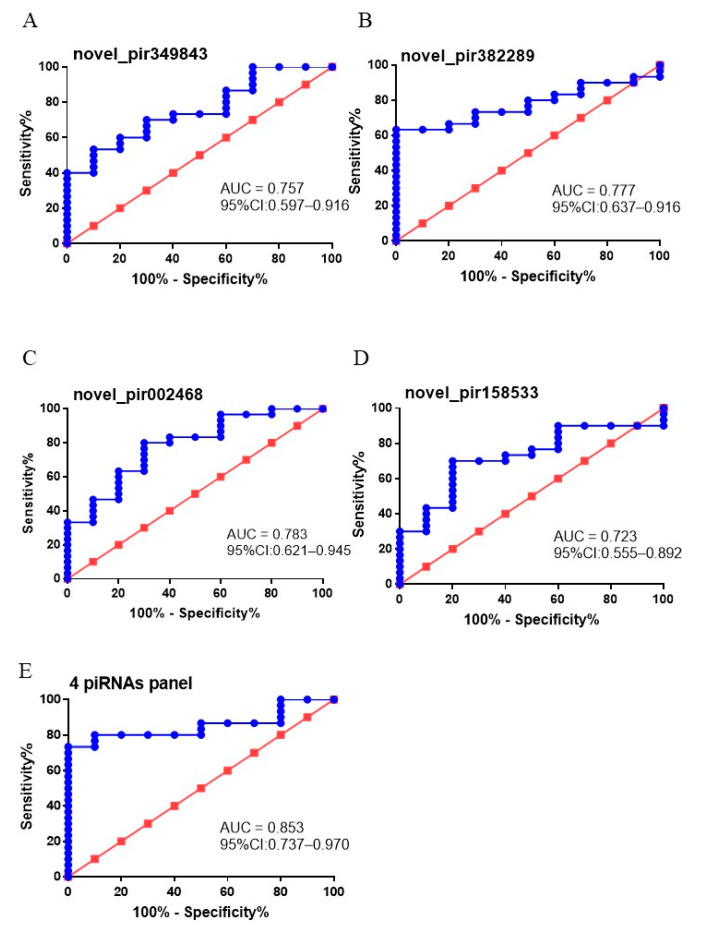
Clinical utility of urinary EVs piRNAs in the diagnosis of PCa. The ROC curves for (**A**) novel_pir349843, (**B**) novel_pir382289, (**C**) hsa_piR_002468, (**D**) novel_pir158533, and (**E**) a combination of the four piRNAs.

To test whether dysregulated urinary EVs novel_pir349843, novel_pir382289, novel_pir158533, and hsa_piR_002468 levels can be used as diagnostic biomarkers for PCa patients, ROC analysis was performed to investigate their diagnostic value for PCa. As shown in Figure 3A–D, AUCs of the four piRNAs to discriminate patients with PCa from patients without PCa ranged from 0.723 to 0.783. Among them, hsa_piR_002468 presented the largest AUC (0.783; 95% CI: 0.621–0.945; sensitivity = 80%, and specificity = 70%) (Figure 3A), followed by novel_pir382289 (0.777; 95% CI: 0.637–0.916; sensitivity = 63.33%, and specificity = 100%) (Figure 3B). In addition, a combination of these four piRNAs was found to further improve the discriminative potential for PCa prediction (AUC 0.853; 95% CI 0.737–0.970) (Figure 3E).

### 3.6. Identification of the Potential Downstream Mechanisms of the Four Dysregulated piRNAs

The RNAhybrid algorithm identified some key genes involved in PCa pathway that had potential binding effect with novel_pir349843, novel_pir382289, novel_pir158533, and hsa_piR_002468. FASTA sequence files of the four piRNAs were obtained from the prediction file according to Piano prediction algorithm. The target binding sites of novel_pir349843, novel_pir382289, novel_pir158533, and hsa_piR_002468 are presented in Table S4. Results suggested that some important gene ontologies and pathways such as “androgen receptor pathway”, “PI3K−Akt-mTOR signaling pathway” might be potential downstream targets of novel_pir158533 and hsa_piR_002468. For novel_pir382289 and novel_pir349843, “Wnt/β-catenin pathway”, “PI3K−Akt-mTOR signaling pathway” and “Ras-Raf-MEK-ERK signaling pathway” might be potential downstream targets.

## 4. Discussion

Urine biomarkers for non-invasive PCa diagnosis have been investigated over the years, and a few have been developed for clinical use including PCA3 [26], SelectMDx [27], and more recently the urine spermine test [28]. EVs carry protein, nucleic acids, etc., and are potential biomarker targets for disease diagnosis and prognosis. In addition, EVs secreted from tumor cells may contain a variety of piRNAs originating from tumor cells, which may be aberrantly expressed in tumor cells [29]. Early evidence showed that piRNAs are involved in PCa development and progression, such as proliferation, apoptosis, metastasis, and invasion [19,20,30]. However, the diagnostic value of piRNA for PCa diagnosis has not been investigated. In this study, we showed for the first time that various urinary EVs piRNA including novel_pir349843, novel_pir382289, novel_pir158533, and hsa_piR_002468 might be potential biomarkers for PCa diagnosis. In addition, the combination of the four piRNAs could further improve the diagnostic value of PCa.

EVs have been traditionally considered as cellular rubbish, a simple means for disposing unnecessary cellular waste products. It was not until the mid-1990s that EVs were gradually exhibited as having a crucial role in intercellular communication, in normal physiological processes, and in the pathogenesis of disease, including cancer [31]. EVs isolated from biofluids of cancer patients have been shown to contain tumor-derived functional molecules, which might be a powerful non-invasive diagnostic and prognostic tool for cancer [32]. However, EVs isolation, especially in urine, can be a challenging procedure. During our preparation work for the study, we had tried different methods of EVs isolation from human urine samples, such as ultracentrifugation at 240,000 g for 1 h, 15% PEG6000 overnight precipitation, Total Exosome Isolation Reagent (Thermo Fisher), and ultrafiltration (Table S5). Among different approaches, ultrafiltration was found to have multiple advantages, including higher reproducibility, shorter processing time, and cost-effectiveness.

Though several studies have identified EVs miRNAs as potential targets for PCa diagnosis, most of these are small-scale, vary in methodology, and lack external validation [22,23,33]. On the other hand, more than 30,000 piRNAs have been identified in the human genome, which is much greater than miRNAs [12], and yet little has been known about piRNAs in PCa. Emerging evidence suggests that in addition to miRNA, piRNAs are also expressed in a tissue-specific manner in a variety of human tissues and modulate key signaling pathways at the transcriptional or post-transcriptional level [10]. Increasing studies have demonstrated that aberrant expression of a number of piRNAs are dysregulated in various cancers, including gastric cancer [34], breast cancer [35], lung cancer [36], colorectal cancer [37], multiple myeloma [38], and bladder cancer [39], which are involved in the tumorigenesis and progression of these tumor types. Additionally, EVs could provide their cargo protection from degradation by cellular RNases, and could be detected in a surprisingly stable form in vivo. Therefore, this makes EVs piRNAs potential candidates for biomarkers, that could provide information of potential pathophysiological conditions, and also influence specific treatment strategies in cancer patients [40].

piRNAs can inhibit target function through binding a diverse spectrum of downstream target genes by forming specific piRNA silencing complexes (pi-RISC), leading to RNA repression via imperfect base-pairing between the two types of RNAs [41], similar to miRNA mechanisms. These RNAs include mRNA [36], transcribed pseudogenes [42], and long non-coding RNA (lncRNA) [43]. Based upon piRNA:mRNA sequence complementarity, we used the RNAhybrid program to search for PCa pathway-related targets of these four significantly upregulated piRNAs against human transcripts with a mean free energy of maximum −20 kcal/mol. The PI3K-Akt-mTOR signaling cascade is frequently activated in PCa, and promotes tumor growth by mediating a plethora of cellular processes [44]. Multiple potential binding sites for novel_pir349843, novel_pir382289, novel_pir158533, and hsa_pir_002468 on the target mRNA of key PTEN-PI3K-Akt-mTOR signaling molecules, which imply the involvement of these piRNA in PCa development. In addition, novel_pir349843, novel_pir382289 also have potential binding sites with the mRNAs of key genes in the Wnt/β-catenin signaling pathway. Androgen receptor (AR) is critical for PCa development and progression at all stages of disease [45]. In this study, AR was identified as a potential target of novel_pir158533 and hsa_pir_002468, which may inspire us to explore the potential mechanism of these four piRNAs in the development of PCa in the future.

As piRNAs function mainly upstream of multiple signaling pathways and regulatory networks [14], it would be interesting to identify PCa-related piRNAs in urinary EVs and identify their potential utility in PCa diagnosis. Urinary EVs piRNAs detection as a non-invasive method may represent promising new complementary tumor markers for the early diagnosis of PCa. In this study, we investigated the EVs piRNA profiles of PCa patients using small RNA sequencing combined with RT-qPCR validation. We identified four EVs piRNAs, including novel_pir349843, novel_pir382289, novel_pir158533, and hsa_piR_002468, that were significantly increased in urinary EVs from PCa patients, in comparison with those from healthy controls by RT-qPCR. These four piRNAs also held relatively high diagnostic accuracy in selecting PCa patients from healthy controls, the AUC of the four piRNA in PCa diagnosis was 0.723, 0.757, 0.777, and 0.783 respectively. For comparison, it has been reported that the AUC for PSA to discriminate between any PCa and cancer-free controls in the Prostate Cancer Prevention Trial was 0.68 (95% CI 0.67–0.69), showing the diagnostic potential of these piRNAs for PCa [46]. A strength of this study includes being the first study to investigate urinary EVs piRNA in PCa diagnosis, the use of RNA sequencing and subsequent PCR validation in another cohort to confirm findings, and the development of a urinary EVs piRNA panel for more accurate PCa diagnosis.

Our study regarding EVs piRNAs has faced some challenges and limitations: (1) Our study sample size is relatively small and is a single-center study, which may need multicenter studies to recruit more patients to further validate the role of EVs piRNAs in PCa diagnosis. (2) The expression level of total RNAs in urinary EVs is low, most of which are below 1ng/uL. RT-qPCR detection reagents with stronger sensitivity and specificity are required, which may increase the cost of potential clinical transformation in the future. (3) The endogenous and exogenous factors that affect the production of EVs have not yet been identified, which, to some extent, complicates the use of EVs piRNAs as clinical biological markers for cancer. Moreover, all patients included in our study are localized PCa with different PCa risk. For those advanced and/or metastatic PCa, high serum PSA levels already show good performance in discriminating healthy from advanced/metastatic PCa. Although EVs piRNA panel combining these four piRNAs showed good performance in discriminating between PCa and healthy controls, they are not suitable as biomarkers for the diagnosis of advanced PCa and metastatic PCa. Further investigations of EVs piRNAs must be performed to determine if they are specifically related to PCa, and explore the underlying molecular mechanisms in PCa.

## 5. Conclusions

Our study showed for the first time that urinary EVs novel_pir349843, novel_pir382289, novel_pir158533, and hsa_piR_002468 were upregulated in PCa patients and may serve as promising biomarkers for PCa diagnosis. A piRNA panel combining these four piRNAs showed good performance in distinguishing PCa from healthy controls.

## Figures and Tables

**Table 1:** Clinical characteristics of patients in the screening and validation cohort.

Variables	Screening Cohort (Small RNA Sequencing)		Validation Cohort (RT-qPCR)	
	PCa Patients (*n* = 5)	Non-PCa Patients (*n* = 5)	PCa Patients (*n* = 30)	Non-PCa Patients (*n* = 10)
Age (years)	69(63–71)	71(64*–*74)	69(64*–*71)	65(62*–*72)
PSA (ng/mL)	7(6.6*–*9.9)	1.55(1.18*–*2.42)	8.7(6.88*–*11.55)	2.33(1.01*–*3.46)
TRUS-PV (ml)	36(27.3*–*44)	N/A	31.5(26.5*–*40.3)	N/A
Clinical T stage		N/A		N/A
cT1	2(40%)		10(33.3%)	
cT2	2(40%)		15(50%)	
cT3	1(20%)		5(16.7%)	
Pathological T stage		N/A		N/A
pT2	1(20%)		12(40%)	
pT3	1(20%)		4(13.3%)	
Gleason score		N/A		N/A
6	4(80%)		15(50%)	
7*–*10	1(20%)		15(50%)	

PCa: prostate cancer; TRUS-PV: prostate volume obtained via transrectal ultrasound; N/A: not available.

**Table 2:** Shortlisted piRNAs in screening cohort and validation cohort.

piRNA ID	Screening Cohort (PCa = 5, Non-PCa = 5)				Validation Cohort (PCa = 30, Non-PCa = 10)	
	Expression (Non-PCa)	Expression (PCa)	Log_2_Ratio (PCa/Non-PCa)	*p _adj_*	Up or Down- Regulate in PCa	*p*
novel_pir99473	1.139	65.734	4.71	0	NS	0.0951
novel_pir349843	0.403	13.49	3.83	3.4527 × 10 ^−155^	Upregulate	0.0151 *
novel_pir382289	0.527	15.854	2.91	1.96084 × 10 ^−87^	Upregulate	0.0084 **
novel_pir99492	0.171	6.326	3.40	2.00497 × 10 ^−46^	NS	0.5279
novel_pir4470	0.001	2.283	7.11	4.94546 × 10 ^−22^	NS	0.3930
novel_pir158533	0.001	4.836	7.92	3.13574 × 10 ^−3^	Upregulate	0.0383 *
novel_pir123744	0.001	2.546	7.00	9.75296 × 10 ^−21^	ND	ND
novel_pir50190	0.001	0.088	4.54	9.67 × 10 ^−5^	NS	0.6335
hsa_piR_001170	5.655	1.356	−2.04	2.26965 × 10 ^−88^	NS	0.1397
hsa_piR_002468	2.033	0.076	−4.69	1.4262 × 10 ^−49^	Upregulate	0.0068 **

*p _adj_*, adjusted *p* values; NS, not significant; ND: not detectable; * statistically significant difference (*p* < 0.05), ** statistically highly significant difference (*p* < 0.001*).*

## Data Availability

Not applicable.

## Supplementary Materials

The following are available online at https://www.mdpi.com/article/10.3390/diagnostics11101828/s1, Figure S1: The expressions of novel_pir99473, novel_pir99492, novel_pir4470, novel_pir50190, and hsa_piR_001170 in validation cohorts. Table S1: Primer sequences for reverse transcription-quantitative polymerase chain reaction analysis. Table S2: Number of total reads, and reads mapped to the genome per sample. Table S3: Summary of detected small non-coding RNA (sncRNA) for each sample. Table S4: The list of piRNAs target genes related with PCa signaling pathways. Table S5: The success rate of EVs RNA extraction under different methods. Table S6: Individual clinical features of HC and PCa patients in two cohorts.
