# Supplementary material for: Identification of piRNA Targets in Urinary Extracellular Vesicles for the Diagnosis of Prostate Cancer
Source: Diagnostics (Basel). 2021 Oct 3;11(10):1828. doi: 10.3390/diagnostics11101828 (PMC8534571; doi:10.3390/diagnostics11101828)
Supplement: Supplementary file 1 [file diagnostics-11-01828-s001.zip › diagnostics-1365443-SI.pdf]

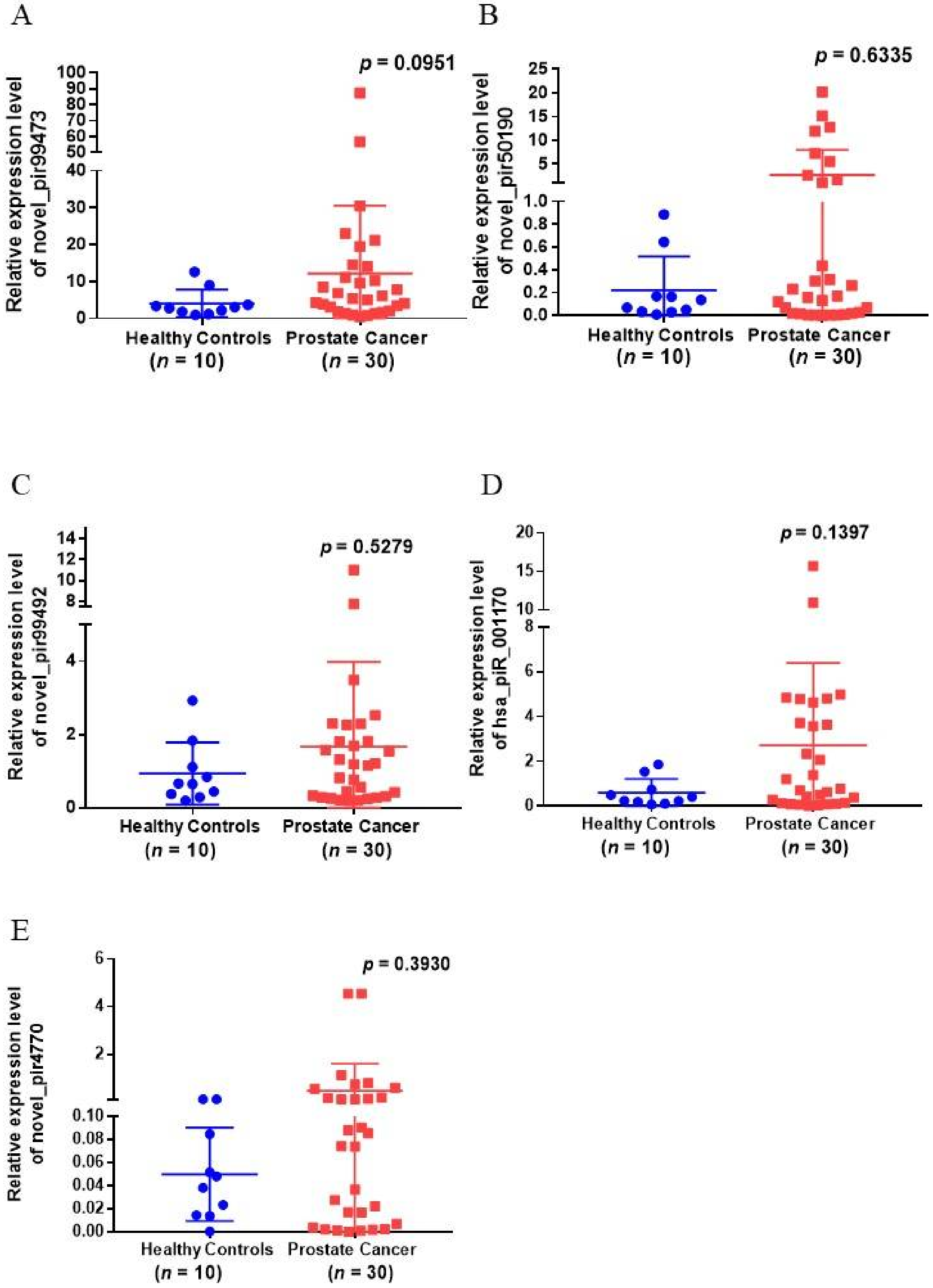

Figure S1. The expressions of novel_pir99473, novel_pir99492, novel_pir4470, novel_pir50190, and hsa_piR_001170 in validation cohorts. (**A**–**E**) Relative piRNA levels of novel_pir99473 (**A**), novel_pir50190 (**B**), novel_pir99492 (**C**), hsa_piR_001170 (**D**) and novel_pir4470 (**E**) in urinary EVs of PCa patients (*n* = 30) and healthy controls (*n* = 10) were investigated by qRT-PCR.

Table S1. Primer sequences for reverse transcription-quantitative polymerase chain reaction analysis.

Name	Sequence (5′-3′)
novel_pir99473<br>novel_pir349843<br>novel_pir382289<br>novel_pir158533<br>novel_pir99492<br>novel_pir50190<br>novel_pir4770<br>novel_pir123744<br>hsa_piR_001170<br>hsa_piR_002468<br>snU6	GCTCACACCTGTAATCCCAGCACTTTGGG<br>CTGAGGCAGGAGAATTGCTTGAACCTGGG<br>CTGAGGCAGGAGAATCACTTGAACCTGGG<br>TCTAATAGGGGTCCATGCTCCGTCTC<br>TCACACCTGTAATCCCAGCACTTTGGG<br>TCCTCTTTGCCTTCTGCCATGATTGTAAGT<br>TTGAAGAGGTCCTTCACGTCCCTTGTA<br>ACAACTGAGTGCTAATGTCGTCTAGA<br>ATAGGTTTGGTCCTAGCCTTTCTATTAGCTCT<br>TCAGAAGATTCCAGGTTCGACTCCTGG<br>GTGCTCGCTTCGGCAGCACATATACTAAAAT<br>TGGAACGATACAGAGAAGATTAGCATGGCCC<br>CTGCGCAAGGATGACACGCAAATTCGTGAAG<br>CGTTCCATATTTT

Table S2. Number of total reads, and reads mapped to the genome per sample.

Sample Name	Total Tag	Mapped Tag	Percentage (%)
N1	20215587	16788307	83.05
N2	24358924	19467563	79.92
N3	22711052	19713651	86.8
N4	26115591	19353199	74.11
N5	22605472	21688961	95.95
T1	23966853	21822634	91.05
T2	25617658	22247645	86.84
T3	20552966	18035370	87.75
T4	23807970	23066238	96.88
T5	20650510	18545425	89.81

Table S3. Summary of detected small non-coding RNA (sncRNA) for each sample.

Sample Name	Known miRNA Count	Novel miRNA Count	Total miRNA Count	Known piRNA Count	Novel piRNA Count	Total piRNA Count	Known siRNA Count	Novel siRNA Count
N1	375	627	1002	156	8187	8343	0	0
N2	345	2345	2690	188	31410	31598	0	0
N3	285	1997	2282	166	18166	18332	0	0
N4	343	288	631	133	13565	13698	0	0
N5	404	5002	5406	121	39643	39764	0	0
T1	373	2361	2734	153	21731	21884	0	0
T2	344	452	796	195	2903	3098	0	0
T3	358	5300	5658	221	47709	47930	0	0
T4	307	7258	7565	194	60572	60766	0	0
T5	406	4478	4884	182	44399	44581	0	0

Table S4. The list of piRNAs target genes related with PCa signaling pathways.

piRNA id	Target Gene Name	RNAhybrid MFE
novel_pir382289	MYC	-21.3
novel_pir382289	GSK3B	-29.6
novel_pir382289	AKT1	-32.9
novel_pir382289	FOXO1	-35.8
novel_pir382289	PIK3CA	-26.6
novel_pir382289	KRAS	-20.1
novel_pir382289	MAP2K7	-20.9
novel_pir349843	MYC	-20.3
novel_pir349843	GSK3B	-30.3
novel_pir349843	PTEN	-23.3
novel_pir349843	AKT1	-33.2
novel_pir349843	FOXO1	-33.3
novel_pir349843	PIK3CA	-28.6
novel_pir349843	KRAS	-22.5
novel_pir349843	MAP2K7	-22.4
novel_pir158533	AR	-23.7
novel_pir158533	GSK3B	-22.3
novel_pir158533	mTOR	-20.9
novel_pir158533	PIK3C2B	-21.7
hsa_piR_002468	AR	-27.4
hsa_piR_002468	PTEN	-20.2
hsa_piR_002468	mTOR	-27.1
hsa_piR_002468	FOXO1	-25.8
hsa_piR_002468	PIK3C2B	-28.8

MFE: Minimum free energy.

Table S5. The success rate of EVs RNA extraction under different methods.

Methodology	Cases Amount	RNA Concentration in Successful Cases (ng/μL)	Successful Rate (Success/Fail)
Exosome isolation kit + Trizol LS	8	1.27 - 3.37	37.5% (3/5)
Exosome isolation kit + NucleoSpin	4	0.48	25% (1/3)
Frozen samples + Trizol LS	6	0.44 - 1.1	33% (2/4)
Fresh samples + Trizol LS	6	0.884 – 1.34	50% (3/3)
Frozen samples, ultracentrifugation + Trizol LS	5	0.526 - 9	40% (2/3)
Fresh samples, ultracentrifugation + Trizol LS	6	1.61	16.7% (1/5)
Frozen samples, 15% PEG6000, ultracentrifugation + Trizol LS	5	0.55 – 1.8	100% (5/0)
Fresh samples, 15% PEG6000, ultracentrifugation + Trizol LS	33	0.4 - 20	63.6% (21/12)

Table S6. Individual clinical features of HC and PCa patients in two cohorts.

Screening Cohort	Diagnosis	AGE (Yeas)	PSA (ng/mL)	TRUS-PV (mL)	Clinical T Stage	Gleason Score
N1	Benign	59	1.16	N/A	N/A	N/A
N2	Benign	69	1.55	N/A	N/A	N/A
N3	Benign	71	2.57	N/A	N/A	N/A
N4	Benign	71	2.26	N/A	N/A	N/A
N5	Benign	77	1.2	N/A	N/A	N/A
T1	PCa	67	8.6	25	T3a	3+3
T2	PCa	60	6.5	34	T1c	3+4
T3	PCa	70	6.7	38	T1c	3+3
T4	PCa	69	11.2	46	T2a	3+3
T5	PCa	72	7	N/A	T2a	3+3
**Validation Cohort**	**Diagnosis**	**AGE (Yeas)**	**PSA (ng/mL)**	**TRUS-PV (mL)**	**Clinical T Stage**	**Gleason Score**
N6	Benign	70	0.6	N/A	N/A	N/A
N7	Benign	70	0.68	N/A	N/A	N/A
N8	Benign	60	1.34	N/A	N/A	N/A
N9	Benign	64	2.1	N/A	N/A	N/A
N10	Benign	61	2.33	N/A	N/A	N/A
N11	Benign	82	3.12	N/A	N/A	N/A
N12	Benign	65	3.3	N/A	N/A	N/A
N13	Benign	62	3.61	N/A	N/A	N/A
N14	Benign	62	3.85	N/A	N/A	N/A
N15	Benign	76	N/A	N/A	N/A	N/A
T6	PCa	68	4.7	37.3	T1c	3+3
T7	PCa	78	5.1	30	T1c	3+3
T8	PCa	63	5.5	27	T1c	3+3
T9	PCa	70	7.1	101	T1c	3+3
T10	PCa	71	7.4	41	T1c	3+3
T11	PCa	71	7.9	32	T1c	3+3
T12	PCa	72	8	18	T1c	3+3
T13	PCa	76	9.3	28	T1	3+3
T14	PCa	59	9.8	25	T1c	3+3
T15	PCa	71	13.9	36	T1c	4+4
T16	PCa	75	1.9	9.3	T2c	3+3
T17	PCa	79	5.1	30	T2c	4+5
T18	PCa	69	6.5	31	T2	3+4
T19	PCa	66	7	60	T2c	3+3
T20	PCa	75	7.7	40	T2b	4+5
T21	PCa	67	8.1	46	T2	3+3
T22	PCa	67	8.6	25	T2a	3+3
T23	PCa	59	8.8	25	T2b	3+3
T24	PCa	69	9.9	40	T2c	3+4
T25	PCa	63	10.3	43	T2	3+4
T26	PCa	69	11	73	T2	3+4
T27	PCa	64	11.2	46	T2	3+4
T28	PCa	64	13	33.3	T3a	4+5
T29	PCa	71	16.7	27	T2c	3+4
T30	PCa	68	17.3	27	T2b	3+4
T31	PCa	64	5.9	32.6	T3a	3+3
T32	PCa	70	10.6	22.9	T3a	3+4
T33	PCa	61	12.6	40	T3b	3+4
T34	PCa	67	13.6	27.8	T3b	4+5
T35	PCa	72	17.1	24	T2c	3+4
